# Hemodialysis-induced positional changes in lamina cribrosa

**DOI:** 10.1038/s41598-024-65700-2

**Published:** 2024-07-02

**Authors:** Ji Hong Kim, Hyo Chan Jeong, Yong Un Shin, Won June Lee

**Affiliations:** 1 Department of Ophthalmology, Hanyang University College of Medicine, 222-1, Wangsimni-ro Seongdong-gu, Seoul, 104763 Korea; 2 Department of Ophthalmology, Hanyang University Seoul Hospital, 222-1, Wangsimni-ro Seongdong-gu, Seoul, 104763 Korea; 3 Department of Ophthalmology, Hanyang University Guri Hospital, Guri, Korea

**Keywords:** Anatomy, Diseases, Nephrology

## Abstract

This study is aimed to investigate the effect of hemodialysis (HD) on the lamina cribrosa (LC) of the optic nerve head (ONH) using swept-source optical coherence tomography (SS-OCT) and other ophthalmological parameters in patients with end-stage kidney disease (ESKD). This prospective observational study included 29 patients who underwent HD for ESKD. ONH parameters including neural canal diameter (NCD), peripapillary vertical height (PVH), and anterior LC depth (LCD), were assessed using SS-OCT. Changes in the ONH parameters before and after HD were statistically analysed. Correlations between changes in the LCD and other ocular and systemic measurements were identified using Pearson’s correlation analyses. The mean anterior LCD significantly decreased from 441.6 ± 139.8 μm before HD to 413.5 ± 141.7 μm after HD (*P* = 0.001). Mean NCD and PVH did not show significant changes after HD (*P* = 0.841 and *P* = 0.574, respectively). A significant correlation was found between changes in the anterior LCD and the mean ocular perfusion pressure (r = 0.397, *P* = 0.036). We observed a significant decrease in anterior LCD after HD. Our study suggests that HD can influence the ONH, especially in the LC.

## Introduction

Chronic kidney disease (CKD) is characterized by a permanent reduction in the glomerular filtration rate and irreversible renal dysfunction. When the glomerular filtration rate drops below 15 mL/min/1.73m^2^, it signifies end-stage kidney disease (ESKD), necessitating renal replacement therapy such as dialysis or transplantation. Hemodialysis (HD), a commonly employed renal replacement therapy, aims to restore fluid balance and eliminate uremic substances by removing excess fluid^1^. However, HD-induced dynamic changes in body fluid composition can lead to various systemic effects, including reduced body weight and systolic blood pressure (SBP)^2,3^. In addition to these systemic alterations, HD reportedly impacts the eye in several ways.

The ocular effects of HD have garnered considerable attention due to the shared pathophysiological mechanisms and potential interplay between the eyes and kidneys^4,5^. Patients with ESKD undergoing HD often experience a range of ocular manifestations, including refractive changes, dry eye, increased tear osmolarity, conjunctival calcium deposits, band keratopathy, lenticular opacities, corneal endothelial changes, and alterations in intraocular pressure (IOP)^6–8^. In addition, thickness changes in the retina and choroid have also been reported^8–12^. Notably, the prevalence of ocular diseases is higher among individuals with CKD than among those without CKD, raising intriguing questions regarding the association between CKD, ESKD, and glaucoma^13–19^. Although research findings on this association are conflicting, numerous clinical and epidemiological studies have investigated the potential relationship between CKD and glaucoma^15,18–20^.

The lamina cribrosa (LC) is a barrier between intraocular and cerebrospinal fluid (CSF) spaces. The difference between IOP and CSF pressure is called the trans-LC pressure difference (TLCPD) and is thought to play an important role in the development of glaucoma^21,22^. When the IOP is higher than the CSF pressure, the LC is pushed backwards compared to normal^23–25^. Conversely, elevated ICP leads to anterior displacement of the LC^26^. Mechanical strain on the LC and subsequent backward shifting can manifest in individuals with glaucoma, potentially resulting in damage to the optic nerve head (ONH), which encompasses the axons of ganglion cells^27,28^. Meanwhile, an increase in ICP has been reported in approximately 60% of patients after renal replacement therapy, including HD^29,30^. Ultimately, it is assumed that HD affects both IOP and ICP, and that the position of the LC can change depending on the interaction of these two pressures.

Several studies have examined acute changes in the retina, choroid, and anterior chamber angle before and after HD using swept-source optical coherence tomography (SS-OCT)^31–33^. Most recently, we reported a decrease in peripapillary choroidal thickness (PCT) immediately after HD^34^. Although these studies can suggest several structural changes after HD, they cannot fully address the association between ESKD and glaucoma. Furthermore, no study has examined changes in the LC in relation to glaucoma. Therefore, this study aimed to investigate the acute effect of HD on the LC of the ONH using SS-OCT and other ophthalmologic parameters in patients with ESKD.

## Results

### Demographics and baseline clinical characteristics of the study participants

Table 1 presents the demographic, baseline ocular, and systemic characteristics of the patients. This study included 29 patients with ESKD undergoing HD (12 men and 17 women). The mean age of the patients was 55.7 ± 10.4 years, with 72.4% having hypertension and 51.7% having diabetes. The mean ultrafiltration (UF) volume was 2.9 ± 0.8 L.

**Table 1:** Demographics and baseline clinical characteristics of the study subjects.

Characteristics	Numbers (n = 29)
Age (years)	55.7 ± 10.4
Sex (male:female)	12:17
Hypertension	21 (72.4%)
Diabetes	15 (51.7%)
IOP (mmHg)	17.3 ± 3.1
AXL (mm)	23.2 ± 1.2
Body weight (kg)	62.5 ± 10.9
SBP (mmHg)	151.2 ± 31.6
DBP (mmHg)	76.6 ± 11.5
MABP (mmHg)	101.5 ± 11.9
MOPP (mmHg)	56.1 ± 7.8
UF (L)	2.9 ± 0.8

*AXL* axial length; *DBP* diastolic blood pressure; *HD* hemodialysis; *IOP* intraocular pressure; *MOPP* mean ocular perfusion pressure; *SBP* systolic blood pressure; *UF* ultrafiltration.

### Comparison of ocular and systemic measurement before and after hemodialysis

Table 2 presents the changes in ocular and systemic measurements before and after HD. IOP showed no significant change from 17.3 ± 3.1 mmHg before HD to 17.1 ± 3.0 mmHg after HD (*P* = 0.603). Body weight exhibited a significant decrease from 62.5 ± 10.9 kg before HD to 59.9 ± 11.0 kg after HD (*P* < 0.001). Furthermore, there was a significant reduction in SBP from 151.2 ± 31.6 mmHg to 140.4 ± 28.8 mmHg (*P* = 0.016), and mean ocular perfusion pressure (MOPP) from 56.1 ± 7.8 mmHg to 54.9 ± 10.1 mmHg (*P* = 0.016). In the OCT measurements, there was a significant increase in retinal nerve fibre layer (RNFL), ganglion cell complex (GCC), and ganglion cell-inner plexiform layer (GCIPL) thicknesses (*P* < 0.001, *P* = 0.003, and *P* = 0.001, respectively), whereas macular choroidal thickness (MCT) exhibited a significant decrease (*P* < 0.001).

**Table 2:** Comparison of ocular and systemic measurement before and after hemodialysis.

Characteristics	Before HD	After HD	Differences	*P*-value
Ocular measurements				
IOP (mmHg)	17.3 ± 3.1	17.1 ± 3.0	− 0.2 ± 2.6	0.603
AXL (mm)	23.2 ± 1.2	23.5 ± 1.4	0.3 ± 1.0	< 0.001
Systemic measurements				
Body weight (kg)	62.5 ± 10.9	59.9 ± 11.0	− 2.6 ± 0.8	< 0.001
SBP (mmHg)	151.2 ± 31.6	140.4 ± 28.8	− 10.9 ± 21.2	0.016
DBP (mmHg)	76.6 ± 11.5	78.9 ± 11.1	2.3 ± 14.0	0.305
MABP (mmHg)	101.5 ± 11.9	99.4 ± 11.5	− 2.1 ± 13.8	0.420
MOPP (mmHg)	56.1 ± 7.8	54.9 ± 10.1	− 1.3 ± 9.6	0.016
OCT measurements				
RNFL (μm)	96.8 ± 15.2	99.4 ± 15.1	2.5 ± 3.1	< 0.001
MRT (μm)	252.8 ± 14.4	255.2 ± 15.3	2.4 ± 7.6	0.101
GCC (μm)	95.6 ± 11.1	96.5 ± 11.7	0.9 ± 4.2	0.003
GCIPL (μm)	64.7 ± 7.4	65.2 ± 7.8	0.5 ± 2.4	0.001
MCT (μm)	220.2 ± 72.8	209.0 ± 74.0	− 11.2 ± 13.7	< 0.001
ONH parameters				
NCD (μm)	1653.5 ± 243.7	1650.8 ± 230.6	− 2.7 ± 71.1	0.841
PVH (μm)	324.8 ± 113.9	321.5 ± 107.1	3.3 ± 30.2	0.574
LCD (μm)	441.6 ± 139.8	413.5 ± 141.7	28.1 ± 37.8	0.001

*AXL* axial length; *DBP* diastolic blood pressure; *GCC* ganglion cell complex; *GCIPL* ganglion cell-inner plexiform layer; *HD* hemodialysis; *IOP* intraocular pressure; *LCD* lamina cribrosa depth; *MABP* mean arterial blood pressure; *MCT* macular choroidal thickness; *MOPP* mean ocular perfusion pressure; *MRT* macular retinal thickness; *NCD* neural canal diameter; *OCT* optical coherence tomography; *ONH* optic nerve head; *PVH* papillary vertical height; *RNFL* retinal nerve fiber layer; *SBP* systolic blood pressure.

Data are mean ± standard deviation.

Comparisons were performed with the paired *t* test.

### Comparison of ONH parameters before and after hemodialysis

**Figure 1:**
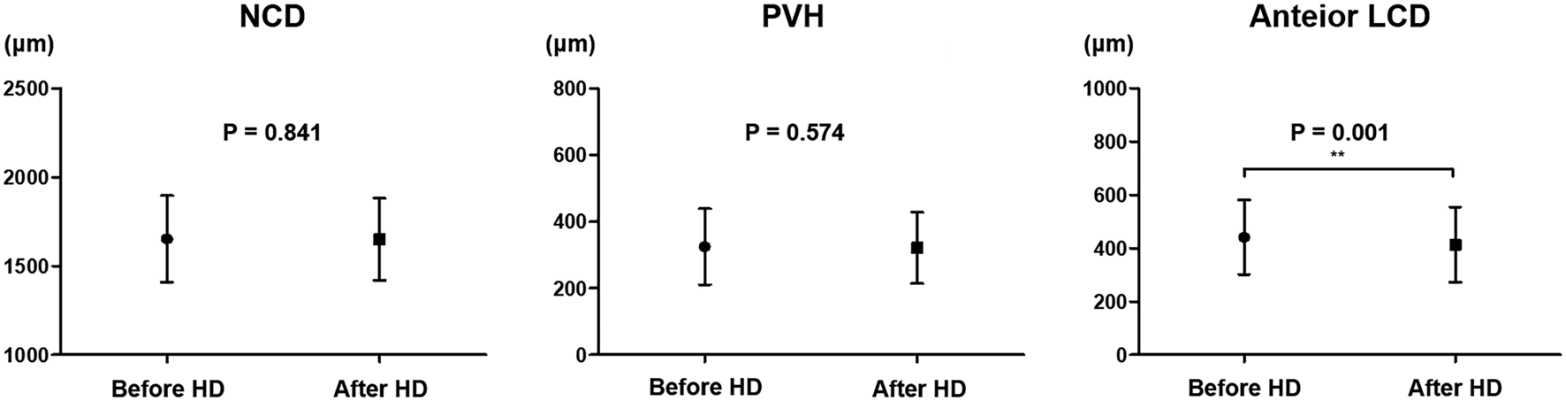
Changes in neural canal diameter (NCD), papillary vertical height (PVH), and anterior lamina cribrosa depth (LCD) before and after hemodialysis (HD).

Table 2 and Fig. 1 present the changes in ONH parameters before and after HD. Neither neural canal diameter (NCD) nor papillary vertical height (PVH) showed significant changes after HD (*P* = 0.841 and *P* = 0.574, respectively). However, anterior lamina cribrosa depth (LCD) exhibited a significant decrease from 441.6 ± 139.8 μm before HD to 413.5 ± 141.7 μm after HD (*P* = 0.001).

### Correlation between changes in *lamina* cribrosa depth and changes in other parameters

Table 3 presents the correlation between the changes in LCD and in other parameters. Correlations between changes in anterior LCD before and after HD and alterations in diastolic blood pressure (DBP), mean arterial blood pressure (MABP), and MOPP were observed (*r* = 0.453, *P* < 0.05 for △DBP; *r* = 0.518, *P* < 0.01 for △MABP; and *r* = 0.397, *P* < 0.05 for △MOPP). Notably, among these correlations, MOPP was the only parameter that exhibited significant changes before and after HD.

**Table 3:** Correlation between changes in lamina cribrosa depth and changes in other parameters before and after hemodialysis.

	△LCD	△SBP	△DBP	△MABP	△MOPP	UF	△RNFL	△MRT	△GCC	△GCIPL	△MCT
△LCD	NA	0.216	0.453*	0.518**	0.397*	0.083	0.002	0.155	0.099	0.126	− 0.085
△SBP	0.216	NA	0.310	0.744**	0.717**	− 0.124	0.094	0.013	− 0.118	− 0.074	0.206
△DBP	0.453*	0.310	NA	0.873**	0.862**	− 0.177	0.103	0.073	0.048	0.048	− 0.124
△MABP	0.518**	0.744**	0.873**	NA	0.982**	− 0.154	0.120	0.242	− 0.004	0.070	− 0.032
△MOPP	0.397*	0.717**	0.862**	0.982**	NA	− 0.188	0.119	0.075	− 0.016	0.006	0.030
UF	0.083	− 0.124	− 0.177	− 0.154	− 0.188	NA	− 0.129	0.147	0.194	0.138	0.282
△RNFL	0.002	0.094	0.103	0.120	0.119	− 0.129	NA	− 0.019	0.107	0.053	− 0.146
△MRT	0.155	0.013	0.073	0.242	0.075	0.147	− 0.019	NA	0.863**	0.962**	− 0.066
△GCC	0.099	− 0.118	0.048	− 0.004	− 0.016	0.194	0.107	0.863**	NA	0.862**	− 0.213
△GCIPL	0.126	− 0.074	0.048	0.070	0.006	0.138	0.053	0.962**	0.862**	NA	− 0.134
△MCT	− 0.085	0.206	− 0.124	− 0.032	0.030	0.282	− 0.146	− 0.066	− 0.213	− 0.134	NA

*△* delta; *DBP* diastolic blood pressure; *GCC* ganglion cell complex; *GCIPL* ganglion cell-inner plexiform layer; *LCD* lamina cribrosa depth; *MABP* mean arterial blood pressure; *MCT* macular choroidal thickness; *MOPP* mean ocular perfusion pressure; *MRT* macular retinal thickness; *RNFL* retinal nerve fiber layer; *SBP* systolic blood pressure; *UF* ultrafiltration.

Correlation coefficients are shown. Pearson correlation was used for evaluating correlation.

Boldface values are statistically significant. Among them, underlined values are statistically significantly changed before and after hemodialysis.

*P < 0.05.

**P < 0.01.

## Discussion

Among the ONH parameters measured using SS-OCT before and after HD, a significant decrease in the anterior LCD was observed after HD, indicating the anterior displacement of the LC. The extent of change in LCD exhibited a significant positive correlation with alterations in MOPP. To the best of our knowledge, this is the first study to confirm the acute changes in LC morphology resulting from HD.

The LC, a sieve-like structure in the posterior sclera, serves as the exit pathway for retinal ganglion cell axons and is considered the primary site for glaucomatous damage. According to the mechanical theory associated with glaucoma development, posterior deformation of the LC is a key pathogenic structural change^35^. The LC is susceptible to damage due to its relatively fragile structure compared to the robust surrounding sclera and its location, which is influenced by both IOP anteriorly and intracranial pressure (ICP) posteriorly. Despite numerous studies investigating the effects of HD on ocular parameters, especially IOP, there is a notable lack of studies focusing on LC morphology. Unlike the gradual changes associated with the pathogenesis of glaucoma, which follow a chronic course, we hypothesized that the rapid hemodynamic fluctuations induced by HD could result in acute shifts in the LC position. Considering the high prevalence of glaucoma in ESKD patients and their undergoing HD, it was emphasized the importance of evaluating the morphology of the LC before and after HD, as sudden changes in LC morphology due to HD could lead to glaucomatous optic nerve damage.

From the perspective of TLCPD, rapid changes in the IOP and ICP may result in acute LC deformation. A well-documented scenario wherein LC morphology undergoes acute changes is glaucoma filtration surgery. Numerous studies have consistently reported a decrease in the LCD and an increase in the thickness and flattening of the LC following trabeculectomy due to reduced IOP^36–38^. While our findings aligned with the anterior displacement of the LC, notably, we observed no significant change in IOP. This suggests that HD induces alterations in LC morphology through a mechanism independent of IOP.

Regarding ICP, one reported case suggests that LC position normalisation occurs after surgical compression in a patient with papilledema^26^. This implies that an increase in ICP may push the LC forward, which is consistent with our results. A study reported an increase in ICP after HD in specific patient populations^39^ However, it is important to note that these patients had acute brain injuries, posing challenges in generalising the findings to a broader population undergoing HD. Furthermore, conditions that lead to drastic ICP changes, such as surgical compression or lumbar puncture, differ from typical scenarios encountered in general HD.

Another study reported anterior displacement of the LC after the Valsalva manoeuvre^40^. The increase in intrathoracic pressure during the Valsalva manoeuvre reduces venous return, leading to increased IOP due to choroidal engorgement and increased episcleral pressure. Despite this, the study explained that because the increase in ICP outweighs the rise in IOP, the LC as a whole experiences forward displacement. However, as HD reduces venous return, its mechanism can be considered entirely distinct from that of the Valsalva manoeuvre.

Historically, visualising the posterior location of the LC within the sclera has posed challenges. However, the advent of SS-OCT, with its capacity to penetrate deeply using long wavelengths, has enabled comprehensive imaging of the ONH, including the LC and peripapillary choroid^41,42^. In our study, among various ONH parameters, we measured NCD, PVH, and anterior LCD using BMO as a reference point. The BMO marks the termination of the retinal pigment epithelial layer on OCT and indicates the position of the optic disc^43^. It was anticipated that alterations in NCD would accompany significant changes in ONH morphology induced by HD. However, our study observed no change in the NCD before and after HD, suggesting that the rapid changes occurring within a few hours due to HD did not significantly impact the relatively static position of the BMO. PVH can increase in cases of papilledema due to elevated CSF pressure^44,45^. A study examining OCT measurements of papilledema in patients with idiopathic intracranial hypertension found correlations with ICP, particularly with maximum height (similar to PVH)^46^, suggesting that PVH measurements in our study could serve as indirect indicators of ICP. Although direct measurement of ICP was not performed in our study, the absence of significant changes in PVH indirectly suggests that alterations in ICP following HD were not substantial enough to affect ONH morphology.

We noted a decrease in the anterior LCD without significant changes in IOP or, albeit assumptive, without significant changes in ICP. This implies that relying solely on TLCPD is insufficient to account for the anterior displacement of the LC observed after HD. Thus, we focused on changes in MOPP, as it demonstrated a significant decrease after HD. Moreover, there was a positive correlation between the degree of MOPP reduction and the extent of anterior LCD decrease. Similar to our findings, previous studies reported a decrease in MOPP after HD^47^. A decline in the MOPP has the potential to induce ischemia in the tissue surrounding the optic nerve, leading to optic nerve damage. This may form the basis of the vascular hypothesis, which posits that reduced perfusion of the optic nerve is a major factor in the development of glaucomatous damage. A decrease in MOPP may arise from a combination of relatively low BP and high IOP. In this study, the IOP exhibited no significant changes, whereas there was a notable decrease in SBP, which was presumed to contribute to the observed decrease in MOPP. While the BP level itself does not demonstrate a clear relationship with glaucoma^48,49^ it has been established that low MOPP serves as a risk factor for glaucoma^50^. Consequently, the reduction in MOPP observed after HD in this study is a potential risk factor for optic nerve damage.

Therefore, it is crucial to examine the mechanism by which a decrease in the MOPP links to anterior displacement of the LC. To date, no study has directly elucidated the correlation between MOPP and LCD. However, drawing from a previous study that demonstrated a reduction in PCT after HD^34^ we can indirectly infer a potential link between MOPP and LCD. The peripapillary choroid plays a crucial role in supplying blood to ONH. Thus, a decrease in the MOPP is anticipated to result in diminished choroidal perfusion, leading to decreased PCT levels. Given that the measurement of LCD using BMO encompasses choroidal thickness^51^ a decline in PCT may ultimately contribute to a reduction in the anterior LCD. To validate this hypothesis, analysis of blood flow around the optic nerve using optical coherence tomography angiography (OCTA) centred on the disc would be beneficial.

Our study had several strengths. First, it marks the first confirmation of the changes in ONH morphology before and after HD. Notably, visualisation of the LC situated in the posterior sclera using SS-OCT and measurement of the anterior LCD allowed for the unprecedented confirmation of anterior displacement of the LC. Second, in addressing the acute deformation of the LC, our study pioneered an explanation using MOPP rather than the conventional perspectives of IOP, ICP, and TLCPD. Though speculative, the basis for this explanation lies in the observed positive correlation between MOPP and anterior LCD.

Despite these strengths, our study had some limitations. First, the inability to measure ICP directly poses a challenge. Although ICP measurement is crucial for explaining LC displacement in the context of TLCPD, ethical considerations prevent direct ICP measurement. However, we indirectly assessed the impact of ICP using parameters such as PVH, an OCT parameter, and observed that post-HD changes were not significant. Second, direct confirmation of changes in optic nerve blood flow using techniques such as fluorescein angiography, indocyanine green angiography, and OCTA was not performed. Consequently, the correlation between changes in MOPP and anterior displacement of the LC can be explained solely by a decrease in PCT. Nevertheless, direct observation of the optic nerve blood flow through these tests would significantly enhance the verification of our hypothesis. The recent introduction of OCTA presents an opportunity to visualize blood flow status around the ONH, including the peripapillary choroid. Therefore, future studies using OCTA before and after HD are crucial. Third, the focus on LCD alone may be insufficient to fully explain LC morphology. However, a comprehensive assessment incorporating additional LC parameters, such as LC thickness and LC curvature index, would provide a more nuanced understanding of changes in LC morphology. Finally, our study only captured acute changes within a single HD session, and long-term alterations in LC morphology were not investigated. Thus, examining the hypothesis that repeated acute LC deformation may lead to sustained optic nerve damage and contribute to glaucoma requires a longitudinal study.

In conclusion, our study, utilising SS-OCT, marks the first confirmation of the impact of HD on the LC. The established correlation between the decrease in MOPP and anterior displacement of the LC allowed for the formulation of a hypothesis regarding acute LC deformation. These findings offer novel insights into the higher incidence of glaucoma in patients undergoing HD.

## Methods

### Participants

This prospective observational study included 29 patients with ESKD who underwent weekly HD sessions on alternate days at the Dialysis Centre in Hanyang University Guri Hospital in South Korea between May and June 2016. The HD sessions lasted 3–4 h with high-performance dialysers maintaining a blood flow rate of 250 mL/min and a dialysate flow rate of 500 mL/min. The study protocol was approved by the Institutional Review Board of the Hanyang University Guri Hospital (IRB no. 2016–05-005). Informed consent was obtained from all the participants. This study adhered to the tenets of the Declaration of Helsinki for Biomedical Research.

Participants who met the inclusion criteria had a best-corrected visual acuity (BCVA) exceeding 6/60 and possessed SS-OCT images with unremarkable media opacity. The exclusion criteria were retinal diseases, with the exception of mild non-proliferative diabetic retinopathy; a history of glaucoma; baseline IOP > 22 mmHg; recent intraocular surgery or intravitreal injection; axial length (AXL) < 21 mm or > 27 mm; and poor-quality SS-OCT images. In instances where both eyes fulfilled the criteria, the eye with the superior BCVA was selected for analysis.

### Measurements

Ocular and systemic measurements have been described in our previous studies^31–34^. Ocular examinations were performed near the dialysis centre on either Monday or Tuesday based on the patients' session days. Comprehensive ophthalmological examinations, including BCVA, IOP, AXL, and SS-OCT, were conducted within 10 min before and after a single HD session, maintaining consistent time intervals. IOP was measured using Tonopen (Reichert Inc., Depew, NY), and AXL was assessed using IOLMaster500 (Carl Zeiss, Jena, Germany). The systemic pre- and post-HD measurements included body weight, UF, SBP and DBP. UF represents the expected fluid removal during HD. The MABP was calculated as DBP + 1/3 (SBP–DBP), and the MOPP was calculated as 2/3 MABP–IOP^52^.

SS-OCT (DRI-OCT Triton; Topcon Inc., Tokyo, Japan) was used to assess the structural changes in the ONH and LC before and after HD. All patients enrolled in this study underwent a wide-field scan that covered a 12 × 9 mm^2^ area, including both the macular and peripapillary regions. The 12 × 9 mm^2^ scan consisted of 256 B-scans, each incorporating 512 A-scans, resulting in 131,072 axial scans per volume. To ensure consistency in the line scan before and after HD, we obtained scans closely matching the line passing through the optic disc using fundus photography. This method also facilitated locating the scan within the OCT image viewer program (IMAGEnet 6, Version 1.24). Additionally, we overlapped the two B-scans obtained before and after HD to verify alignment with the retinal pigment epithelium line as closely as possible. The RNFL, macular retinal thickness, GCC, GCIPL, and MCT were automatically assessed using integrated automated segmentation software and an OCT image viewer program.

### ONH parameters

**Figure 2:**
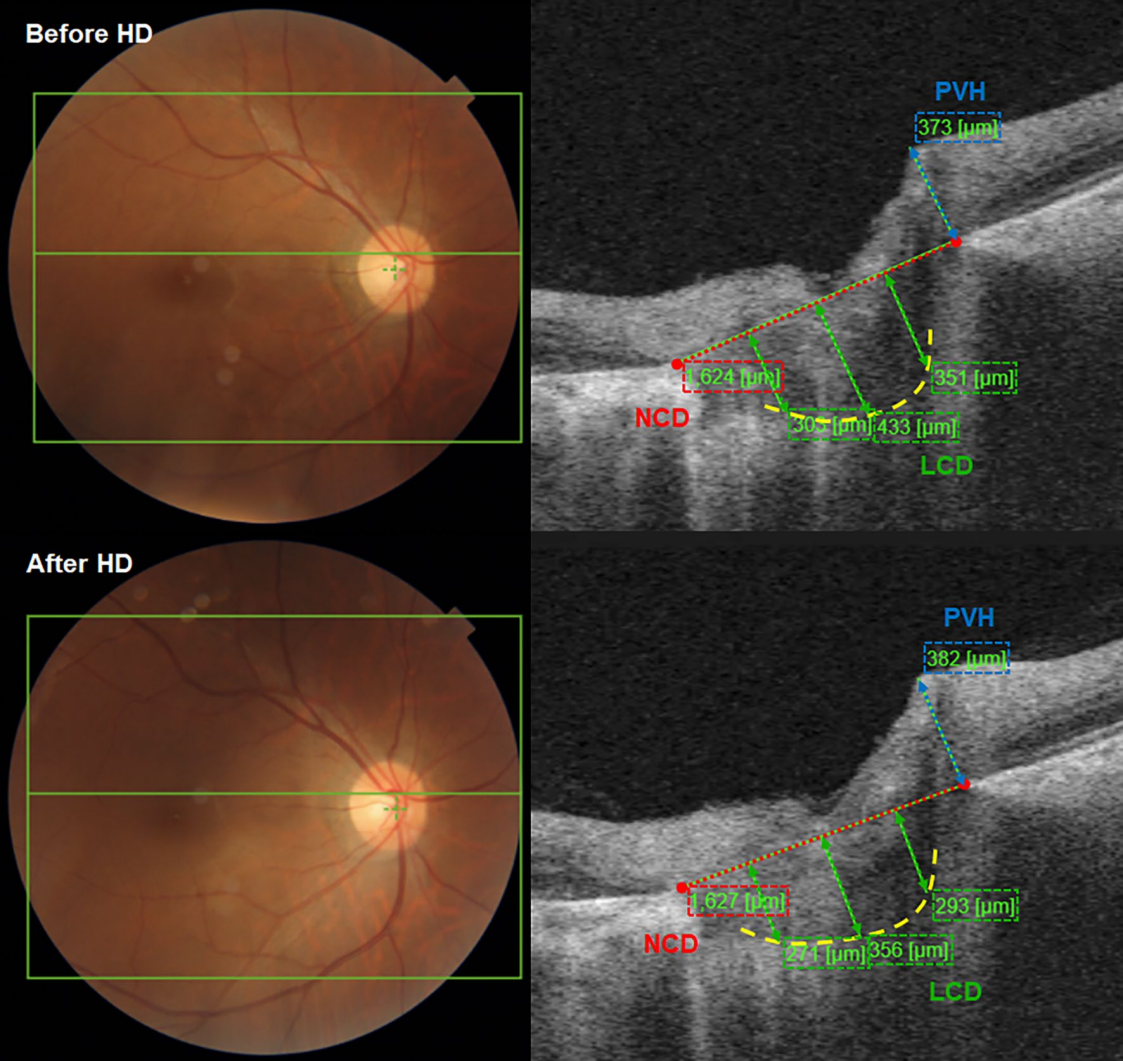
Measurement of neural canal diameter (NCD), papillary vertical height (PVH), and anterior lamina cribrosa depth (LCD) using swept-source optical coherence tomography before and after hemodialysis (HD). The yellow curve represents the anterior border of the LC, the red line indicates the NCD, the blue arrow highlights the PVH, and the green arrow denotes the anterior LCD. The measurement of the anterior LCD involves calculating the average length at the quarter point.

Figure 2 present the representative case showing the changes in ONH parameters before and after HD. On consecutive B-scan images, the section that best showed the anterior border of the LC, including the ONH, within one-third of the mid-periphery of the optic nerve was selected. ONH parameters included the NCD, PVH, and anterior LCD, which were used in the previous report^26^. NCD was defined as the line connecting the Bruch’s membrane opening (BMO), PVH represented the distance from the BMO to the highest point of the internal limiting membrane, and anterior LCD was defined as the distance from the BMO to the anterior surface of the LC. Specifically, the anterior LCD was determined by segmenting the line connecting both the BMOs into quarters. The vertical distances from the three resulting points to the LC were measured using the ruler function embedded in the built-in software and the averages of these distances were subsequently calculated.

### Statistical analysis

Numerical variables are presented as mean ± standard deviation, whereas categorical variables are presented as counts with corresponding percentages. Systemic and ocular measurements, particularly ONH parameters obtained via SS-OCT, were compared before and after HD using a paired t-test. Pearson’s correlation analysis was used to evaluate the correlation between changes in LCD and other ocular and systemic parameters. All statistical analyses were performed using SPSS software version 27 (IBM Corp., Armonk, NY, USA). Statistical significance was set at *P* < 0.05 significant.

## Data Availability

The datasets used and/or analyzed during the current study are available from the corresponding author on reasonable request.
